# Free fatty acid receptors as therapeutic targets for the treatment of diabetes

**DOI:** 10.3389/fphar.2014.00236

**Published:** 2014-11-06

**Authors:** Atsuhiko Ichimura, Sae Hasegawa, Mayu Kasubuchi, Ikuo Kimura

**Affiliations:** ^1^Department of Pharmacogenomics, Kyoto University Graduate School of Pharmaceutical Science, Kyoto, Japan; ^2^Department of Molecular Medicine and Therapy, Tohoku University Graduate School of Medicine, Sendai, Miyagi, Japan; ^3^Department of Applied Biological Science, Graduate School of Agriculture, Tokyo University of Agriculture and Technology, Fuchu-shi, Tokyo, Japan

**Keywords:** free fatty acids, FFAR1, FFAR2, FFAR3, FFAR4, obesity, diabetes

## Abstract

Nutrition regulates energy balance; however, dysfunction of energy balance can cause metabolic disorders, such as obesity and diabetes. Fatty acids are an essential energy source and signaling molecules that regulate various cellular processes and physiological functions. Recently, several orphan G protein-coupled receptors were identified as free fatty acid receptors (FFARs). GPR40/FFAR1 and GPR120/FFAR4 are activated by medium- and/or long-chain fatty acids, whereas GPR41/FFAR3 and GPR43/FFAR2 are activated by short-chain fatty acids. FFARs are regarded as targets for novel drugs to treat metabolic disorders, such as obesity and type 2 diabetes, because recent studies have showed that these receptors are involved in the energy metabolism in various tissues, including adipose, intestinal, and immune tissue. In this review, we summarize physiological roles of the FFARs, provide a comprehensive overview of energy regulation by FFARs, and discuss new prospects for treatment of metabolic disorders.

## INTRODUCTION

Poor dietary habits are a major risk factor for metabolic syndrome. Shifting to a diet low in carbohydrate and high in fat can causes the accumulation of excess energy leading to metabolic disorders, such as obesity, hyperlipidemia, and type 2 diabetes (Kahn et al., 2006; Sanz et al., 2010). In addition to glucose and amino acids, fatty acids derived from dietary fat are an essential energy source for life. Recently, a series of orphan G protein-coupled receptors (GPCRs) were determined to be free fatty acid receptors (FFARs), suggesting that free fatty acids (FFAs) are both energy source and signaling molecules (Hara et al., 2013). These receptors are now regarded as targets for novel drugs for the treatment of metabolic syndromes because they are activated by FFAs. In this review, we summarize the current understanding of the physiological roles of FFARs in energy metabolism and discuss their potential as therapeutic targets for the treatment of obesity and diabetes.

## FATTY ACIDS AND FFAR FAMILY

Fatty acids are categorized by carbon chains length. Short-chain fatty acids (SCFAs) contain fewer than 6 carbons, medium-chain fatty acids (MCFAs) have 6–12 carbons, and long-chain fatty acids (LCFAs) contain more than 12 carbons. Fatty acids are an important energy source and are supplied primarily through food intake, biosynthesis, and lipolysis from adipose tissues. For example, the human body cannot generate α-linolenic acid C18:3 (n-3) and linoleic acid C18:2 (n-6), which have more than two double bonds in their structures. Thus, ω-3 and ω-6 fatty acids must be obtained through the diet to facilitate synthesis. MCFAs and LCFAs are metabolized by β-oxidation, and are utilized as an energy source in various tissues. SCFAs, such as acetate, propionate, and butyrate, are produced via gut microbial fermentation of indigestible polysaccharides, including dietary fiber, and are utilized as energy sources by intestinal epithelial cells and liver.

**FIGURE 1:**
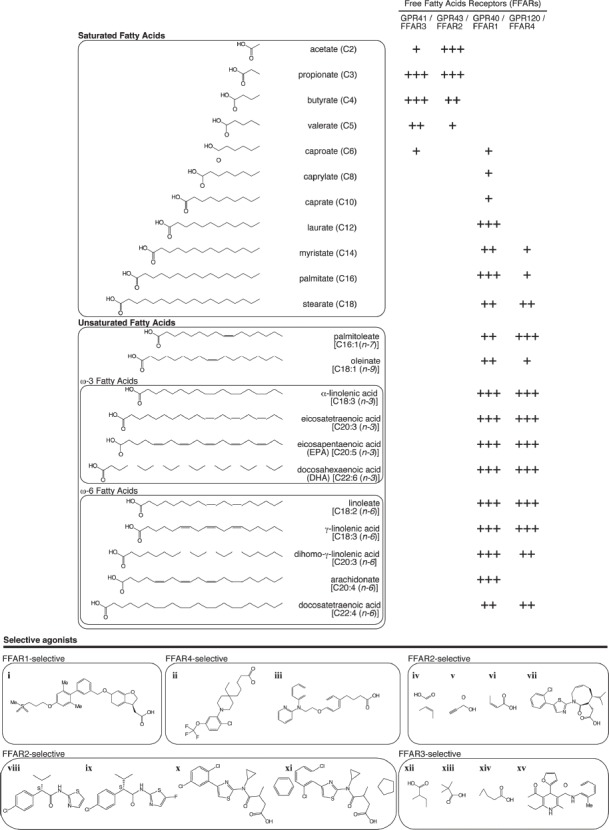
**Affinity of fatty acids and representative selective agonists for free fatty acid receptors (FFARs).** Endogenous (upper panel) and selective [lower panel: **(i)** TAK-875/fasiglifam (Srivastava et al., 2014), **(ii)** CpdA (Oh et al., 2014), **(iii)** NCG-21 (Sun et al., 2010), **(vi)** *trans*-2-methylcrotonic acid, **(v)** propiolic acid, **(vi)** angelic acid (Schmidt et al., 2011), **(vii)** compound 34 (Ulven, 2012; Bindels et al., 2013), **(viii)** MAG7703 phenylacetamide 1, **(ix)** MAG7703 phenylacetamide 2 (Lee et al., 2008), **(x)** compound 1, **(xi)** compound 2 (Hudson et al., 2013), **(xii)** 2-methylbutyric acid, **(xiii)** 1-methylcyclopropanecarboxylic acid, **(xiv)** cyclopropylacetic acid (Schmidt et al., 2011), **(xv)** compound 4 (Leonard et al., 2006)] agonists for FFAR1, FFAR2, FFAR3, and FFAR4.

Recently, it was reported that fatty acids act as both energy sources and as signaling molecules. GPCRs that are activated by FFAs are categorized according to ligand profile, depending on the length of the FFA carbon chain. MCFAs and LCFAs activate FFAR1 and FFAR4, whereas SCFAs activate FFAR3 and FFAR2 (Figure 1; Offermanns, 2014).

## GPR40/FFAR1

FFAR1 is a receptor for MCFAs and LCFAs. Various FFAs activate FFAR1 in the micromolar concentration range, although eicosatrienoic acid C20:3 (n-3) is the most potent agonist of FFAR1 (Itoh et al., 2003). Itoh et al. (2003) reported that LCFAs promote glucose-stimulated insulin secretion (GSIS) in pancreatic β cells. In this report, it was shown that FFAR1 is expressed in pancreatic β cells, and that the specific and acute effects of FFAs on pancreatic β cells were significantly decreased following the loss of FFAR1 function (Itoh et al., 2003). In addition, Steneberg et al. (2005) demonstrated that both the acute and chronic effects of FFAs on insulin secretion were due to activation of FFAR1 using *Ffar1* knockout (KO) mice and pancreatic β cell-specific *Ffar1* transgenic (TG) mice. The chronic effects of FFAs on GSIS were significantly decreased by loss of FFAR1 function. In contrast, TG overexpression of the human *FFAR1* gene in mouse pancreatic β cells under the control of mouse insulin II promoter prevented the development of hyperglycemia in high fat diet (HFD)-fed mice and improved insulin secretion and glucose tolerance in genetically diabetic mice (Nagasumi et al., 2009). Kristinsson et al. (2013) further showed that acute and chronic effects of palmitate on insulin secretion were partly mediated by FFAR1. FFAR1 agonists-induced GSIS was inhibited by the treatment with FFAR1 antagonist. Additionally, long-term treatment with palmitoleate decreased GSIS, and this effect was inhibited by FFAR1 antagonist. Natalicchio et al. (2013) showed that expression of exendin-4 in pancreatic β cells inhibits FFAR1 expression and phosphorylation of stress kinases, such as Jun N-terminal kinase (JNK) and mitogen-activated protein kinase (MAPK), which in turn could prevent the palmitoleate-induced apoptosis. Furthermore, Meidute Abaraviciene et al. (2013) showed that FFAR1 protein level in pancreatic islets are regulated by glucose and FFAs, and are associated with insulin secretion. Repeated administration of selective FFAR1 agonists increased insulin sensitivity and improved glucose metabolism *in vivo* (Tanaka et al., 2013). Thus, several factors, including the energy expenditure, glucose metabolism, plasma FFA level, and expression levels of FFAR1, contribute to the acute chronic effects of FFAs mediated by FFAR1 in pancreatic β cells. FFAR1 is also expressed in intestinal endocrine cells. Edfalk et al. (2008) reported that FFAR1 was expressed in intestinal L and K cells, which secrete the incretin hormones glucagon-like peptide 1 (GLP-1) and glucose-dependent insulinotropic polypeptide (GIP) and in I cells, which secrete cholecystokinin (Liou et al., 2011). Therefore, FFAR1 modulates FFA-induced insulin secretion from β cells directly and indirectly, through the regulation of incretin secretion. Based on this data, a selective FFAR1 agonist might be useful for the treatment of type 2 diabetes. TAK-875, or fasiglifam, developed by Takeda, is an orally available, potent, and selective partial-agonist of FFAR1, which reached phase III clinical trials for the treatment of type 2 diabetes (Srivastava et al., 2014). However, Takeda recently discontinued the phase III trial because of liver toxicity.

## GPR120/FFAR4

FFAR4 was successfully deorphanized in 2005 and identified as a receptor for MCFAs and LCFAs that promotes GLP-1 secretion from the colon (Hirasawa et al., 2005). Various ω-3 or ω-6 polyunsaturated fatty acids, including docosahexaenoic acid (DHA) C22:6 (n-3) and EPA C20:5 (n-3), activate FFAR4 in the micromolar concentration range. Among these FFAs, FFAR4 is most potently activated by α-linolenic acid C18:3 (n-3) (Hirasawa et al., 2005). Although its ligand profiles are similar to those of FFAR1, the amino acid sequence identity between FFAR4 and FFAR1 is only 10%. FFAR4 was shown to be widely expressed in various tissue and cell types, including intestinal tissue, adipose tissue, macrophages, and pancreas (Ichimura et al., 2009, 2012; Oh et al., 2010). The diverse tissue distribution of FFAR4 suggests that FFAR4 has multiple functions in the homeostatic regulation of systemic metabolism and inflammation. FFAR4 is also expressed in adipocytes, but was not detected in pre-adipocytes (Gotoh et al., 2007; Ichimura et al., 2012). Furthermore, FFAR4 expression was increased by lipid accumulation in cells during the induction of adipogenesis in 3T3-L1 cells (Gotoh et al., 2007). Both knockdown of *Ffar4* by siRNA in 3T3-L1 cells and gene deficiency in mouse embryonic fibroblasts suppressed the expression of adipogenic genes and lipid accumulation (Gotoh et al., 2007; Ichimura et al., 2012). These data indicated that FFAR4 may play an important role in the differentiation and maturation of adipocytes. *Ffar4* mRNA expression was increased in white adipose tissue of HFD-fed mice (Gotoh et al., 2007). Recently, we found and reported that gene deficiency and dysfunction of *Ffar4* lead to obesity in both mice and humans (Ichimura et al., 2012). We found that HFD-fed *Ffar4* KO mice developed severe obesity, which was accompanied by decreased differentiation and lipogenesis in adipocytes. Furthermore, severe fatty liver, enhanced hepatic lipogenesis, increased fasting glucose, and impaired responses to insulin and glucose tolerance were observed in HFD-fed *Ffar4* KO mice. We reported two non-synonymous mutations, p.R270H and p.R67C, by exon sequencing of FFAR4 in obese and lean European subjects. Subsequent *in vitro* experiments revealed that the p.R270H mutant, which was significantly associated with obesity, lacked the ability to transduce LCFA signals unlike the p.R67C mutant, which was not associated with obesity. Taken together, the studies of humans lacking functional FFAR4 and *Ffar4* KO mice indicate that dysfunction of FFAR4 leads to obesity in both mice and humans (Ichimura et al., 2012).

Oh and Olefsky (2012) demonstrated that FFAR4 activation exerts anti-inflammatory effects on macrophages. FFAR4 regulates DHA-modulated, lipopolysaccharide (LPS)-induced cyclooxygenase-2 in macrophages through β-arrestin signaling. Thus, FFAR4 is a functional ω-3 FFA receptor that mediates potent insulin sensitizing and antidiabetic effects through suppression of macrophage-induced tissue inflammation. Moreover, Oh et al. (2014) recently reported an orally available small molecule FFAR4 agonist. This selective agonist improved insulin resistance and chronic inflammation in obese mice. Hence, FFAR4 agonists could become future insulin-sensitizing agents for the treatment of type 2 diabetes and other human insulin-resistant states.

## GPR41/FFAR3

FFAR3 is an SCFA receptor that can be activated by propionate (C3), butyrate (C4), and valerate (C5) (Brown et al., 2003; Le Poul et al., 2003). FFAR3 expression in intestinal L cells, which secrete peptides YY (PYY) and GLP-1, indicated that this receptor is involved in energy homeostasis (Tazoe et al., 2009). PYY and GLP-1 secretion was reduced in primary cultured endocrine cells derived from *Ffar3* KO mice (Samuel et al., 2008; Tolhurst et al., 2012). After colonization of germ-free mice with specific microbes, wild type mice, but not *Ffar3* KO mice, showed an increase in PYY levels. Thus, PYY secretion from intestinal L cells is regulated by gut microbes SCFAs acting on FFAR3. We previously reported that *Ffar3* was abundantly expressed in sympathetic ganglia (Kimura et al., 2011). Our data indicated that propionate-induced FFAR3 activation resulted in increased heart rate and energy expenditure via sympathetic outflow. Importantly, these effects were not exhibited in *Ffar3* KO mice. Moreover, sympathetic activation by FFAR3 directly promotes noradrenalin release from sympathetic neurons (Inoue et al., 2012). In contrast, under fasting conditions, FFAR3 contributed to the suppression of energy expenditure. β-Hydroxybutyrate, a ketone body produced in the liver during starvation, decreased sympathetic outflow by antagonizing FFAR3 (Kimura et al., 2011; Inoue et al., 2012). Thus, FFAR3 regulates sympathetic activity by sensing the nutritional state, thereby maintaining body energy homeostasis.

Recently, De Vadder et al. (2014) reported that FFAR3 improves insulin resistance via the gut–brain neural circuit, through activation of peripheral nerve FFAR3 by SCFAs produced from dietary fibers by gut microbes. Thus, FFAR3 agonists acting on peripheral neurons may serve as a novel type of therapeutic for metabolic disorders. Several synthetic compounds were reported as FFAR3 agonists or antagonists and structural analysis revealed that the presence of small carboxylic acids, including cyclopropanecarboxylic acid, in the structure enhanced FFAR3 selectivity (Leonard et al., 2006). Moreover, Hudson et al. (2014) recently reported these synthetic ligands are allosteric modulators of FFAR3 with complex and diverse pharmacology.

## GPR43/FFAR2

FFAR2 is also an SCFA receptor; however, the ligand affinity differs between FFAR2 and FFAR3. Acetate and propionate activate FFAR2 with high potency, followed by butyrate and other SCFAs (Brown et al., 2003; Le Poul et al., 2003). FFAR2 like FFAR3 is expressed in L cells. SCFAs-induced FFAR2 activation promotes GLP-1 secretion in mixed colonic primary cultures and STC-1 cells (Tolhurst et al., 2012; Hudson et al., 2013). *Ffar2* KO mice exhibited reduced SCFA-induced GLP-1 secretion both *in vitro* and *in vivo* and had impaired glucose tolerance. FFAR2 was also abundantly expressed in adipose tissues. Hong et al. (2005) showed that expression of *Ffar2* was significantly greater in white adipose tissues of mice with HFD-induced obesity than in normal chow-fed mice. Further, they showed that SCFAs suppressed isoproterenol-induced lipolysis in a concentration-dependent manner in 3T3-L1-derived adipocytes (Hong et al., 2005). Additionally, Ge et al. (2008) demonstrated that these effects were dependent on FFAR2 using *Ffar2* KO mice.

In a series of *in vitro* and *in vivo* studies using *Ffar2* mutant mice, we showed that SCFA-mediated FFAR2 activation suppressed adipose insulin signaling, leading to inhibition of fat accumulation in adipose tissue (Kimura et al., 2013). In this study, we found that the source of FFAR2 ligands was dependent on gut microbes, because *Ffar2* deficiency induced obesity in mice, whereas mice overexpressing *Ffar2* only in adipose tissues exhibited leanness under normal conditions. Neither of these mouse strains exhibited either phenotype under germ-free conditions or after antibiotic treatment. Based on this data, FFAR2 regulates adipose insulin signaling by sensing SCFAs, provided by gut microbes, thereby regulating fat accumulation and maintaining body energy homeostasis. Thus, FFAR2 stimulation suppresses fat accumulation in adipose tissue and promotes GLP-1 secretion in gut, thereby improving insulin resistance. FFAR2 agonists could be used as novel insulin-sensitizing drugs for the treatment of type 2 diabetes.

Hence, recently, development of FFAR2 selective agonists or antagonists to prevent its pathophysiological functions is progressing. Structure–activity relationship analysis revealed small carboxylic acids at the orthosteric binding site where ligands with substitute sp(2)-hybridized alpha-carbons showed selectivity for FFAR2. In contrast, ligands with sp(3)-hybridized alpha-carbons showed selectivity for FFAR3 (Schmidt et al., 2011). In addition, several selective synthetic FFAR2 agonists have been reported, including allosteric compounds (Lee et al., 2008) and orthosteric compounds (Hudson et al., 2013). Beside FFAR2 related metabolic regulation, many studies have investigated the role of FFAR2 in regulating inflammatory responses (Maslowski et al., 2009; Offermanns, 2014). GLPG0974 developed by Galapagos, is an orally available small FFAR2 inhibitor that reached phase II clinical trials for the potential treatment of inflammatory bowel disease. According to the company, the first exploratory phase II clinical study in ulcerative colitis (ClinicalTrials.gov Identifier^1^: NCT01829321) suggested that “GLPG0974 was safe and well tolerated and showed two relevant biomarker effects.” However, these biomarker reductions did not result in a clinical improvement within 4 weeks^2^.

## CONCLUSION

**FIGURE 2:**
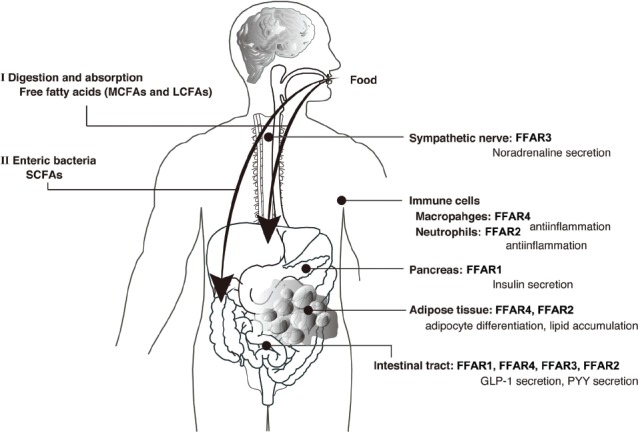
**Physiological functions of free fatty acid receptors (FFARs).** Medium- and long-chain fatty acids derived from dietary fat act as ligands for FFAR1 or FFAR4 **(I)**. Short-chain fatty acids produced by gut microbes from indigestible dietary fiber act as ligands for FFAR3 or FFAR2 **(II)**.

In recent years, many metabolic and immune response pathways have been reported in relation to nutrient sensing systems. Several studies have provided evidence that FFARs are dietary sensors expressed in both metabolic tissues and immune cells that regulate both energy metabolism and inflammatory responses. These investigations will lead to new insights in nutritional science and drug discovery science, due to the importance of FFA signaling in diverse metabolic process (Figure 2). Since the inflammatory response is also related to the development of obesity and type 2 diabetes, the FFAR family is expected to be a potential therapeutic target for the treatment of these diseases. Although the detailed signaling mechanism and physiological functions of FFARs in tissues are unclear, a further understanding of the regulation of energy metabolism and inflammatory responses by the FFAR family represents an important avenue of research in drug development for the treatment of obesity and type 2 diabetes.

### Conflict of Interest Statement

The authors declare that the research was conducted in the absence of any commercial or financial relationships that could be construed as a potential conflict of interest.

## Footnotes

1 http://www.clinicaltrials.gov/ct2/show/NCT01829321?term=GLPG0974&rank=3

2 http://www.glpg.com/index.php/randd/pipeline/glpg0974-inflammatory/
